# Steady-State Kinetics of Enzyme-Catalyzed Hydrolysis of Echothiophate, a P–S Bonded Organophosphorus as Monitored by Spectrofluorimetry

**DOI:** 10.3390/molecules25061371

**Published:** 2020-03-17

**Authors:** Irina V. Zueva, Sofya V. Lushchekina, David Daudé, Eric Chabrière, Patrick Masson

**Affiliations:** 1 Arbuzov Institute of Organic and Physical Chemistry, Federal Research Center “Kazan Scientific Center of the Russian Academy of Sciences”, Arbuzov str. 8, 420088 Kazan, Russia; zueva.irina.vladimirovna@gmail.com; 2 Emanuel Institute of Biochemical Physics, Russian Academy of Sciences, Kosygin str 4, 119334 Moscow, Russia; sofya.lushchekina@gmail.com; 3 Gene&GreenTK, HU Méditerranée Infection, Jean Moulin Blvd 19–21, 13005 Marseille, France; david.daude@gene-greentk.com; 4 Aix-Marseille University, IRD, APHM, MEPHI, IHU-Méditerranée Infection, 15005 Marseille, France; eric.chabriere@univ-amu.fr; 5 Kazan Federal University, Neuropharmacology Laboratory, Kremlevskaya str 18, 480002 Kazan, Russia

**Keywords:** P–S bonded organophosphorus agents, echothiophate, Calbiochem Probe IV, organophosphate hydrolase, phosphotriesterase, cholinesterase, QM/MM

## Abstract

Enzyme-catalyzed hydrolysis of echothiophate, a P–S bonded organophosphorus (OP) model, was spectrofluorimetrically monitored, using Calbiochem Probe IV as the thiol reagent. OP hydrolases were: the G117H mutant of human butyrylcholinesterase capable of hydrolyzing OPs, and a multiple mutant of *Brevundimonas diminuta* phosphotriesterase, GG1, designed to hydrolyze a large spectrum of OPs at high rate, including V agents. Molecular modeling of interaction between Probe IV and OP hydrolases (G117H butyrylcholinesterase, GG1, wild types of *Brevundimonas diminuta* and *Sulfolobus solfataricus* phosphotriesterases, and human paraoxonase-1) was performed. The high sensitivity of the method allowed steady-state kinetic analysis of echothiophate hydrolysis by highly purified G117H butyrylcholinesterase concentration as low as 0.85 nM. Hydrolysis was michaelian with *K_m_* = 0.20 ± 0.03 mM and *k_cat_* = 5.4 ± 1.6 min^−1^. The GG1 phosphotriesterase hydrolyzed echothiophate with a high efficiency (*K_m_* = 2.6 ± 0.2 mM; *k_cat_* = 53400 min^−1^). With a *k_cat_/K_m_* = (2.6 ± 1.6) × 10^7^ M^−1^min^−1^, GG1 fulfills the required condition of potential catalytic bioscavengers. quantum mechanics/molecular mechanics (QM/MM) and molecular docking indicate that Probe IV does not interact significantly with the selected phosphotriesterases. Moreover, results on G117H mutant show that Probe IV does not inhibit butyrylcholinesterase. Therefore, Probe IV can be recommended for monitoring hydrolysis of P–S bonded OPs by thiol-free OP hydrolases.

## 1. Introduction

Organophosphorus agents (OP) are among the most toxic chemicals. These compounds have been used for decades as pesticides or drugs, and the most dreadful are banned chemical warfare agents. They primarily inhibit acetylcholinesterase (AChE) and butyrylcholinesterase (BChE), causing a major cholinergic syndrome [1,2].

**Scheme 1:**
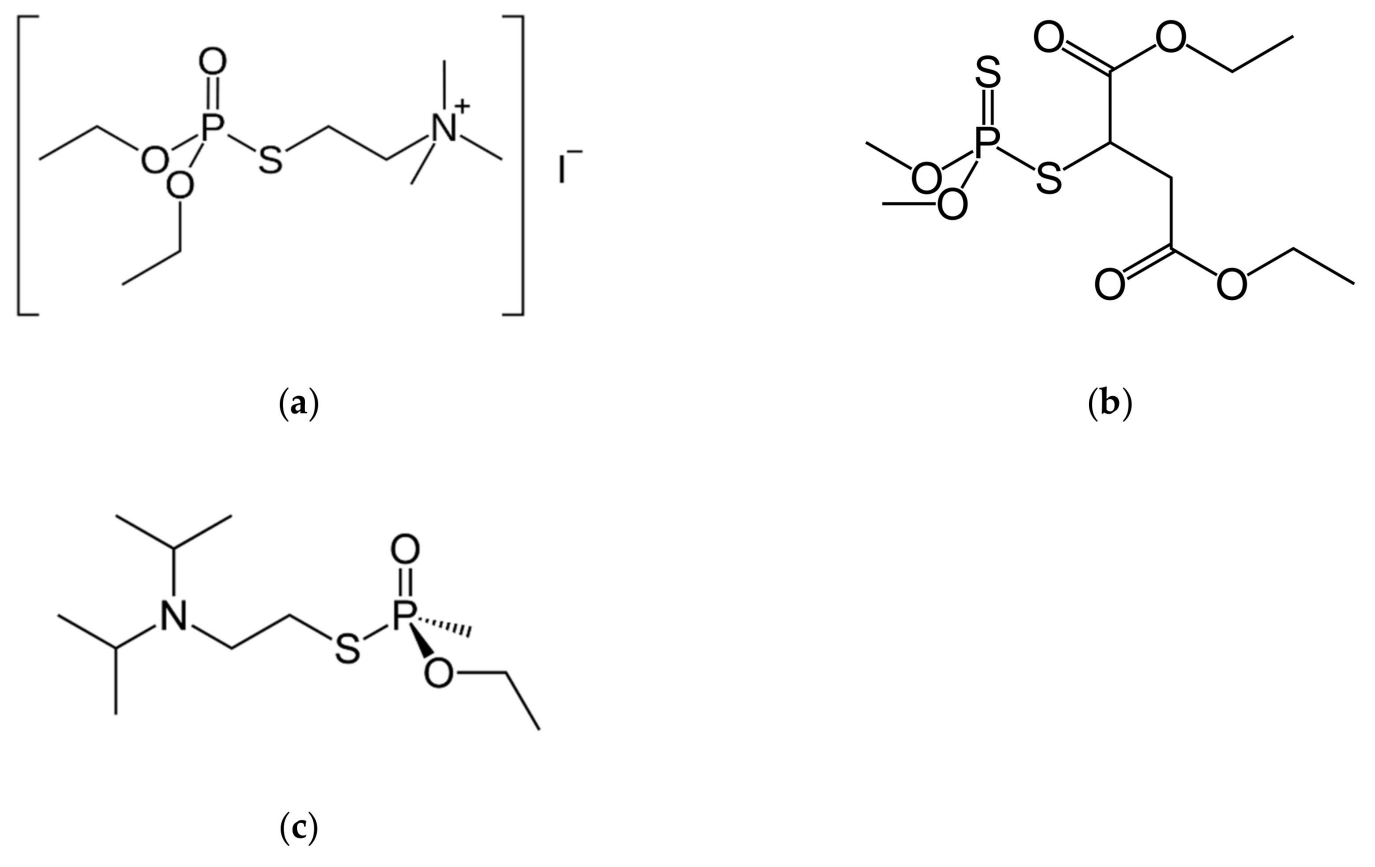
Structure of three P–S bonded OPs: (**a**), echothiophate iodide; (**b**) malathion; (**c**) *S*_p_(-)VX ([2-(diisopropylamino)ethyl]-O-ethyl methylphosphonothioate). Compound “a”, a phosphorothioate, was used as a P–S bonded model OP in the present study.

Several classes of OPs can be described according to atoms/chains bonded to the phosphorus atom. Among them, P–S bonded phosphoro- and phosphono-thioates are of interest (Scheme 1).

For example, echothiophate (phospholine iodide) is a drug used for treatment of glaucoma [3], malathion (carbophos) is an agriculture pesticide for eradication of mosquitos also used for treatment of pediculosis [4,5], and VX is a potent chemical warfare nerve agent [6]. Spontaneous hydrolysis or enzyme-catalyzed hydrolysis of phosphoro/phosphono-thioates and phosphylation of ChEs by these compounds results in breakage of the P–S bond in the P–S-alkyl chain with release of thiol leaving group. There are two fundamentally different mechanisms for enzyme-catalyzed hydrolysis of OPs, for ChE mutants and phosphotriesterases (PTE) [7]. Because ChEs are serine hydrolases, OP hydrolysis catalyzed by ChE mutants involves a two chemical-step mechanisms, as for hydrolysis of carboxylesters: after formation of the productive enzyme-substrate complex the nucleophilic attack of the catalytic serine leads to formation of the phosphyl-intermediate accompanied by release of the oxo- or thio-leaving group, and then water-mediated hydrolysis of this intermediate takes place. On the other hand, PTEs are metalloenzymes and the hydrolysis mechanism involves only one chemical step. Although intimate mechanisms of PTE are still debated, there is a consensus, stating that hydrolysis results from a direct attack on P atom of a water-derived hydroxyl group bridging metal cations [8].

Therefore, hydrolytic degradation of phosphoro/phosphono-thioates can be monitored by measuring the rate of formation of the thiol leaving group. There are numerous chromogenic thiol probes, one of the most widely used is the Ellman’s reagent, DTNB, (5,5′-dithiobis-2-nitro) benzoic acid, that releases a yellow anion, 5-thio-2-nitrobenzoate, upon reaction with thiol compounds. This reaction is implemented in the most popular method for assay of ChEs with thiocholine esters as substrates [9]. Because hydrolysis of echothiophate releases also thiocholine (cf. Scheme 1, a), this reaction has been used to monitor the hydrolysis of this OP by the G117H mutant of human BChE and by the mutant HQF of *Bungarus fasciatus* AChE, mutants capable of hydrolyzing OPs [10,11].

**Scheme 2:**
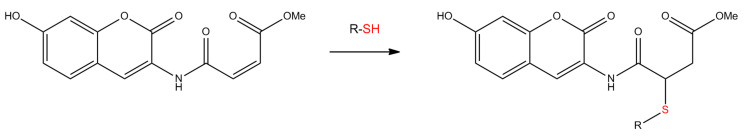
Reaction of Probe IV with a thiol compound (R-SH) giving a highly fluorescent conjugate.

However, rather high concentrations of enzymes are needed for kinetic analysis of P–S bonded hydrolysis of OPs. A fluorogenic thiol probe would be more sensitive than DTNB. The use of the fluorogenic Calbiochem Probe IV ((3-(7-Hydroxy-2-oxo-2H-chromen-3-ylcarbamoyl) acrylic acid methylester) (Scheme 2) was proven to be 2 orders of magnitude more sensitive than the Ellman assay for kinetic analysis of ChEs-catalyzed hydrolysis of thiocholine esters [12].

In the present work, we report steady-state kinetic analysis of degradation a P–S-bonded OP by two OP hydrolases, using Probe IV as thiol probe. OP hydrolases are of great biotechnological and medical interest. Phosphotriesterases (PTE) of various origins, in particular, can be used for detection of OPs [13], decontamination and remediation [8,14,15,16,17,18] and for therapy of OP poisoning as catalytic bioscavengers [7,19,20,21,22]. Because OPs are hemi-substrates of ChEs, research of novel human ChE mutants capable of hydrolyzing OPs is of great interest for improving catalytic bioscavenger-based medical countermeasures of OP poisoning [23,24,25] and implementation of pseudocatalytic bioscavenger systems composed of ChE-reactivator [26,27,28]. Enzymatic hydrolysis of P–S bonded OPs, such as V-agents, is currently monitored by the reaction of the OP thiol leaving group with DTNB [16,29]. However, DTNB is two orders of magnitude less sensitive than Probe IV. 

The use of Probe IV makes it possible to push the limit of detection of P–S bonded OPs. The P–S bonded model OP in the present study was echothiophate iodide, and the enzymes were the reference G117H mutant of human BChE that hydrolyze OPs, and a new multiple mutant of *Brevundimonas diminuta* PTE, GG1, specially designed for rapid hydrolytic detoxification of V-agents [30].

## 2. Results and Discussion

### 2.1. Interaction of Probe IV With Enzymes

#### Molecular Docking Studies

Molecular docking was used to provide information about possible reversible binding of Probe IV to ChEs in our previous work [12]. However, molecular docking techniques have intrinsic limitations [31]. In particular, in the case of ChEs, due to the complex architecture of the deep active site gorge, direct transformation of docking binding affinities into in inhibition constants should be used with caution. In the case of metallo-PTEs, a similar warning has to be pointed out. Nevertheless, molecular docking often provides useful clues about molecular interactions and it is helpful for comparative studies, as in the present report. Furthermore, there is a body of docking studies about numerous ligands into ChEs, coupled with experimental measurements, serving refinement of this approach [32,33]. Also, substantial efforts have been made to parametrize metal-containing systems [34,35] to provide adequate docking score.

**Figure 1:**
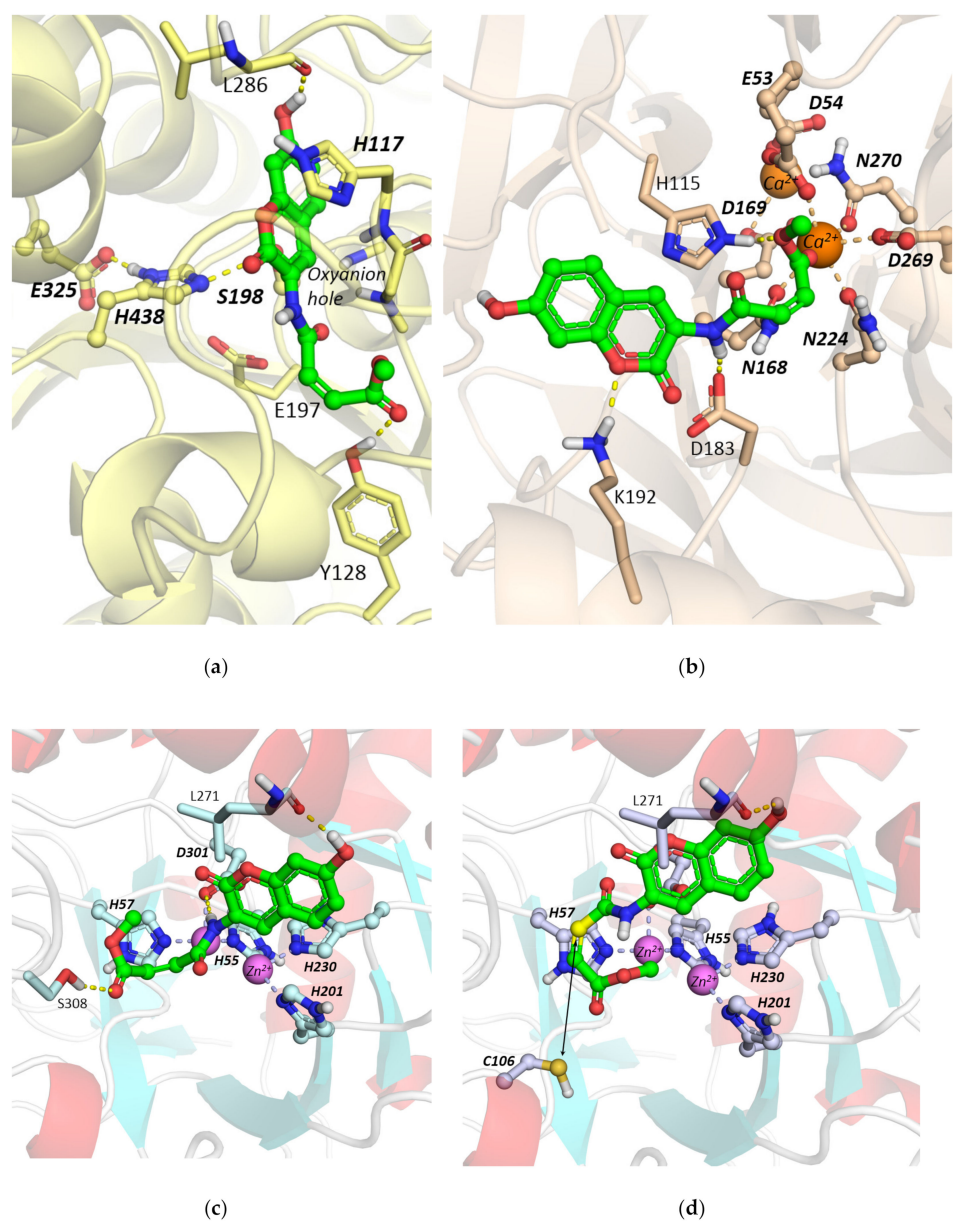

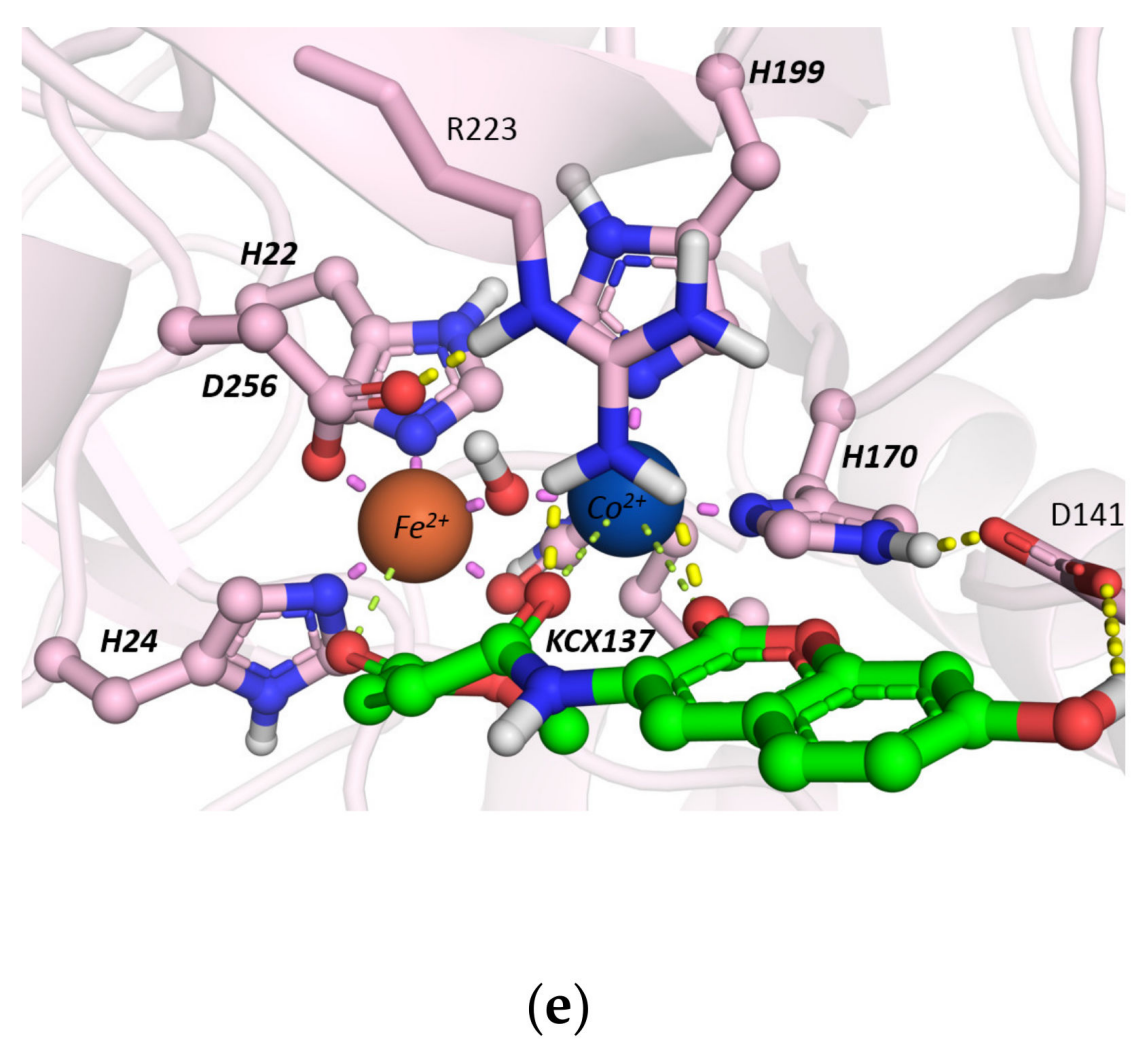
Docked positions of Probe IV inside active sites of (**a**) G117H butyrylcholinesterase (BChE), (**b**) PON-1, (**c**) wt *B. diminuta* phosphotriesterases (PTE), and (**d**) GG1 mutant of *B. diminuta* PTE. In panel (**d**) reacting atom of Probe IV is highlighted yellow and (**e**) *S. solfataricus* PTE.

Molecular docking showed that Probe IV can bind to active sites of all considered enzymes. For human BChE, mutation G117H slightly alters the position of Probe IV in the BchE active site (Figure 1a compared to the wild-type enzyme [12]. This causes a moderate increase of estimated weak binding affinity (−5.76 kcal/mol vs. −6.61 kcal/mol). Thus, we may consider that 1 μM Probe IV does not inhibit G117H.

Very close docked binding affinities were obtained for Probe IV with PON-1 (−6.75 kcal/mol) (Figure 1b) and wild-type *Brevundimonas diminuta* PTE (−6.41 kcal/mol). However, for the PTE mutant GG1 binding was much weaker (−4.16 kcal/mol). Comparison of binding position of Probe IV in active sites of wild-type and GG1 *B. diminuta* PTE (Figure 1c,d) shows that in the latter case Probe IV forms fewer hydrogen bonds. This explains drop in estimated binding free energies. Thus, inhibition of these PTEs would certainly be significant above 10–20 μM Probe IV.

Reaction between Probe IV and Cys106 in GG1 was found to be unlikely, though possible: the minimal distance between sulfur atom and reacting atom of Probe IV among all docked poses was 3.8 Å. This is too long for covalent reaction, but could be got over due to thermal motions.

Binding position of Probe IV in the active site of *Sulfolobus solfataricus* PTE with QM/MM-derived point charges (Figure 1e,f) was closer to position in *B. diminuta* PTE, but binding affinity was weaker (−5.34 kcal/mol). Docking with formal charges +2 on metals shows the importance of accurate parameterization of bi-cations in active sites (without this step, binding affinities were strongly overestimated: −13.4 kcal/mol for *B. diminuta* PTE and −17.10 kcal/mol for *S. solfataricus* PTEs).

### 2.2. Steady-State Kinetics of Enzyme-Catalyzed Hydrolysis of Echothiophate

#### 2.2.1. Hydrolysis of Echothiophate by the G117H Mutant of Human Butyrylcholinesterase

**Figure 2:**
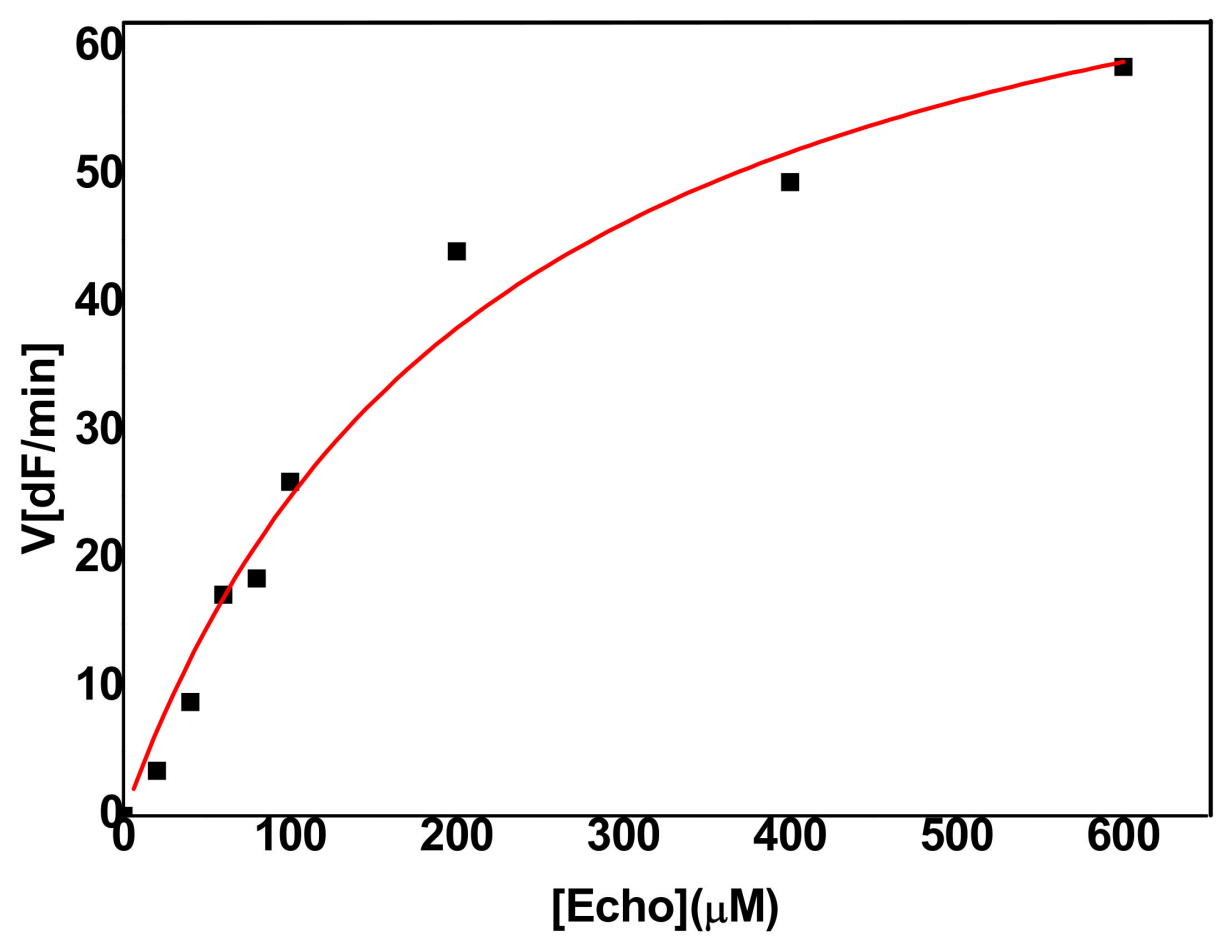
Steady state kinetics of echothiophate hydrolysis by the G117H mutant of human BChE ([E] = 8.5 nM) in 0.1M phosphate buffer pH 7.0 at 25 °C. Fluorescence change/min was converted in micromoles of released thiol/min, using calibration plot established in proper buffer.

Steady-state kinetics analysis of G117H-catalyzed hydrolysis of echothiophate up to 0.6 mM (Figure 2), under pseudo-first order conditions ([E]<<[OP]) was interpreted in terms of Michaelis–Menten model. However, at higher concentration, binding of a second echothiophate molecule on the enzyme peripheral anionic site would be expected, thus, altering the reactivity of the catalytic center as do positively charged substrates and OPs at high concentration [36,37].

The calculated catalytic parameters are: *K_m_* = 0.20 ± 0.03mM, *k_cat_* = 5.4 ± 1.6 min^−1^, and *k_cat_/K_m_* = 2.7 ± 1.6 × 10^4^ M^−1^min^−1^. Though slightly higher, these values are in accordance with previously reported parameters for this mutant with echothiophate under the same conditions: *K_m_* = 0.074 mM, *k_cat_* = 0.75 min^−1^, and *k_cat_/K_m_* = 1.01 × 10^4^ M^−1^ min^−1^ [10]. It should be noted that these old results were obtained, using a partially purified enzyme (<90% pure) at enzyme concentration of 3 × 10^−6^ M per assay. The high sensitivity of Probe IV allowed us to determine the catalytic parameters of G117H with an enzyme more than three–four orders of magnitude less concentrated (0.85–28.5 × 10^−9^ M).

**Figure 3:**
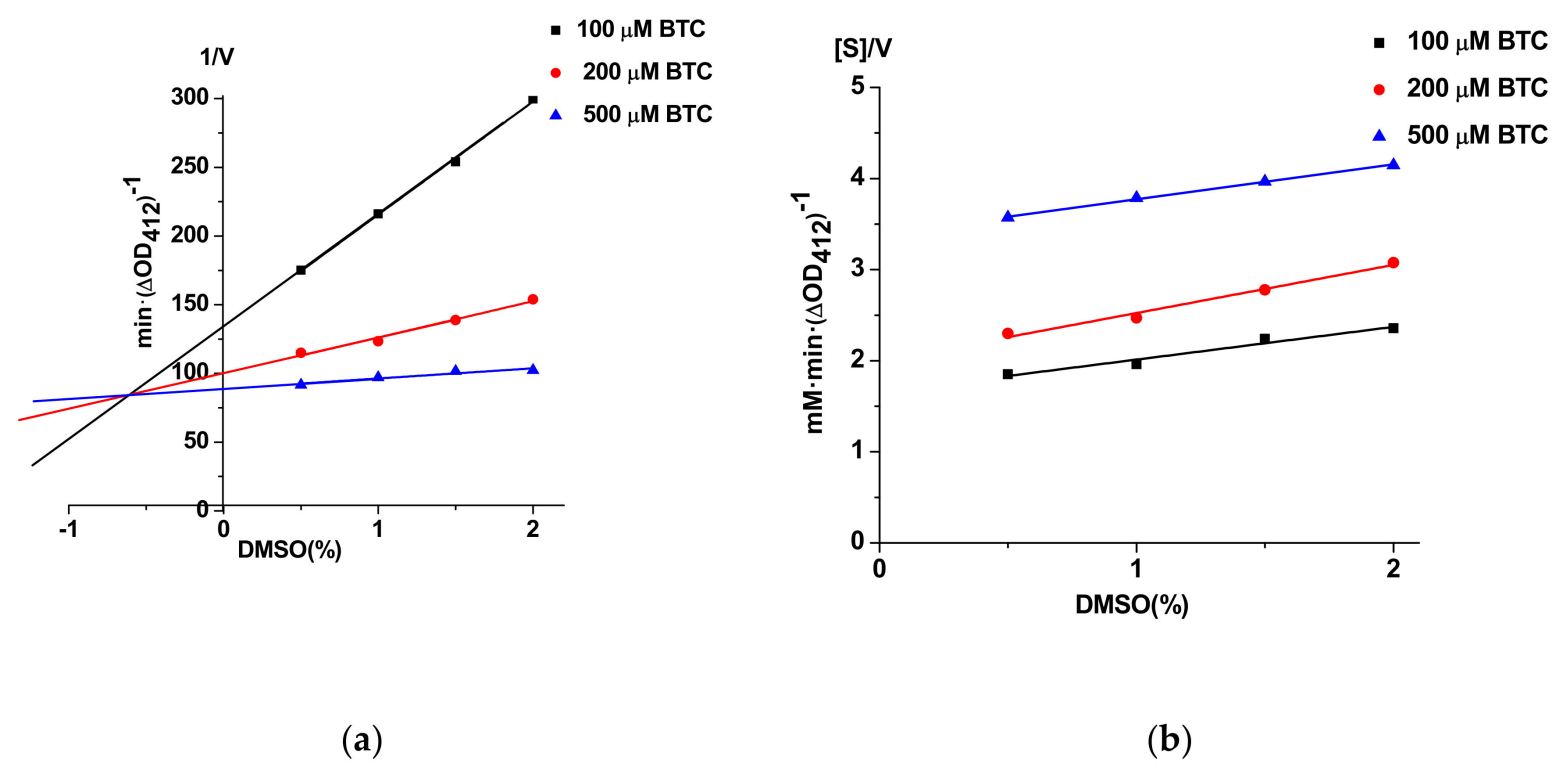
Reversible inhibition of G117H by DMSO, (**a**), Dixon plot; (**b**), Cornish-Bowden plot.

However, our results showed that 1% dimethylsulfoxide (DMSO) inhibits the enzyme. This is in accordance with previously reported data on AChE and BChE [12,38]. Because solvent inhibiting effects are independent on the nature of substrate, the effect of DMSO on G117H was investigated, using the classical Ellman’s method with butyrylthiocholine (BTC) as the substrate. Thus, it was possible to compare the present results on G117H mutant with results previously obtained on wild type and other mutants of human BChE [12,38]. Kinetic analysis of DMSO-induced inhibition of G117H activity toward BTC showed that DMSO is a weak reversible competitive inhibitor with *K*_i_ = 0.6%, i.e., 76 mM (Figure 3). Thus, 1% DMSO (= 128 mM) leads to a *K*_m_ increment 1 + [I] / *K_i_* = 2.68. This value is consistent with the observed 2.7-times increase of apparent *K_m_* of G117H for echothiophate, and three-times increase of apparent *K_m_* of wild-type BChE and E197G and E197D mutants for BTC [12]. Also, the fact that weak reversible inhibition of the enzyme by DMSO fully explains the slight increase in *K_m_* of our results compared to previously reported results [10], indicates that ProbeIV does not compete with echotiophate in G117H enzyme active site.

#### 2.2.2. Hydrolysis of Echothiophate by the GG1 Mutant of Brevundimonas Diminuta PTE

**Figure 4:**
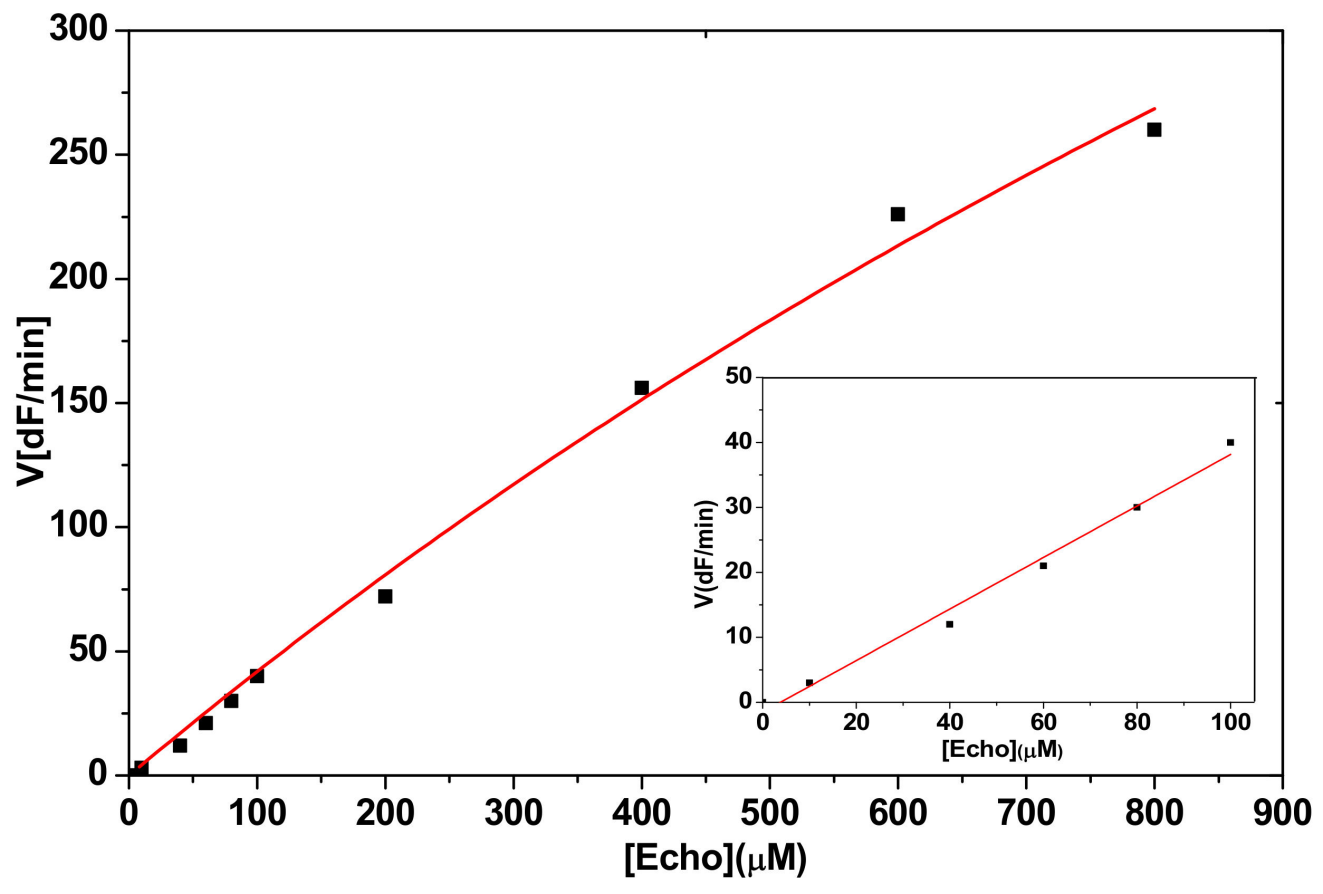
Steady-state GG1-catalyzed hydrolysis of echothiophate. [E] = 0.024 μM. Fluorescence change/min was converted in micromoles of released thiol/min, using calibration plot established in proper buffer.

The GG1 mutant of *Br. d.* PTE was found to be highly effective to hydrolyze echothiophate with *K_m_* = 2.6 ± 0.2 mM; *k_cat_* = 55400 min^−1^and *k_cat_/K_m_* = (2.1 ± 1.6) × 10^7^ M^−1^min^−1^ (Figure 4). These values are in agreement with reported values for related multiple mutants of *Br. d*. PTE with other P–S bonded OPs.

Moreover, assay of the enzyme with 500 μM echothiophate in the presence of various concentrations of DMSO (1%, 1.5%, 2%, and 2.5%) showed no activity change. This indicates that the catalytic activity of GG1 is not altered by the presence of DMSO up to 2.5% (not shown).

Although no data are available with echothiophate for other *Br.d.* PTE mutants that cleave P–S bond, it was reported by Raushel’s group that different multiple mutants of this enzyme capable of hydrolyzing P–S bonded pesticides and V-agents display similar properties, showing the highest specificity constants for malathion: 1 × 10^7^ M^−1^min^−1^ [16], *S*_p_(VX): 5.1 × 10^7^ M^−1^min^−1^, and *S*_p_ (VR): 1.2 × 10^7^ M^−1^min^−1^ [8]. These reported kinetic values were determined by using diluted enzymes whose concentrations typically ranged between 8 × 10^−8^ M (for the most active mutants) and 3 × 10^−5^ M (for wt) [39]. Thus, with a high specificity constant (1.8 × 10^7^ M^−1^min^−1^) for echothiophate as the substrate, the multiple mutant GG1 almost fulfill the minimal required condition (*k_cat_/K_m_* > 5 × 10^7^ M^−1^min^−1^) to be considered as a potential catalytic bioscavenger [7,19].

This statement has important practical therapeutic applications, regardless galenic formulation and safety issues. Indeed, even in the most severe cases of poisoning the OP concentration in blood is extremely low (e.g., ≈ 3 × 10^−6^ M), in vivo enzymatic detoxification of OP by a catalytic bioscavenger operates under first order conditions, according to Equation (3), with the first order rate constant equal to (*k_cat_/K_m_*).[*E*], and:

(1) $$[OP{]}_{t}=[OP{]}_{0}\exp\left(-\frac{k_{\mathrm{cat}}{\left[E\right]}_{0}}{K_{m}}\cdot t\right).$$

Thus, to drop a concentration in echothiophate of 3 × 10^−6^ M by 100-fold (ln[OP]_0_/[OP]_t_ = 4.6) in human blood in 1min (median time for a full turn of blood circulation), an amount of 9 mg of GG1 would be needed to inject to a human of 70 kg. Such a dose is about 20-fold lower than the equivalent dose that would be needed with a stoichiometric bioscavenger like human BChE [40,41]. Decontamination of biological samples or fragile materials by GG1 would also be very effective and fast. Although PTE-based prophylactic and post-exposure treatments would require further developments, including encapsulation in nanoparticles and pharmacokinetics studies, such an enzymatic variant is of prime interest for external decontamination (e.g., skin, materials) for limiting poisoning effects of OPs in humans and environment. On the other hand, the specificity constant (*k_cat_/K_m_*) of ChE-based mutants capable of self-reactivation after phosphylation would have to be increased by four orders of magnitude to reach the >5 × 10^7^ M^−1^min^−1^ condition. This challenge has already been pointed out [24]. However, progress in directed evolution and computer re-design of enzymes make possible to take up the challenge.

## 3. Materials and Methods

### 3.1. Chemicals

Echothiophate iodide was from Biobasal AG (Basel, Switzerland). Calbiochem Probe IV (3-(7-Hydroxy-2-oxo-2H-chromen-3-ylcarbamoyl) acrylic acid methylester) was from Merck Millipore (Darmstadt, Germany). Probe IV is a light sensitive chemical. Glutathione (GSH) used for calibration curve was purchased from Sigma-Aldrich (Saint Louis, MO, USA). All other chemicals were of biochemical grade.

### 3.2. Enzymes

G117 H mutant of human BChE: Low glycosylated and truncated monomeric recombinant G117H mutant of human BChE (MW = 70,000) was produced by O. Lockridge in Chinese hamster ovary (CHO) cells and highly purified for X ray structure determination [42]. The enzyme appeared 100% pure on SDS-4-30% polyacrylamide gradient gel; it was concentrated to 6 mg/mL ([E] = 0.85 × 10^−4^ M) in 10 mM MES pH 6.5, containing 0.02% sodium azide. The enzyme was stored for years at −80 °C without loss of ChE activity as assayed with 1 mM butyrylthiocholine iodide as the substrate (200 unit/mg) [30].

**Figure 5:**
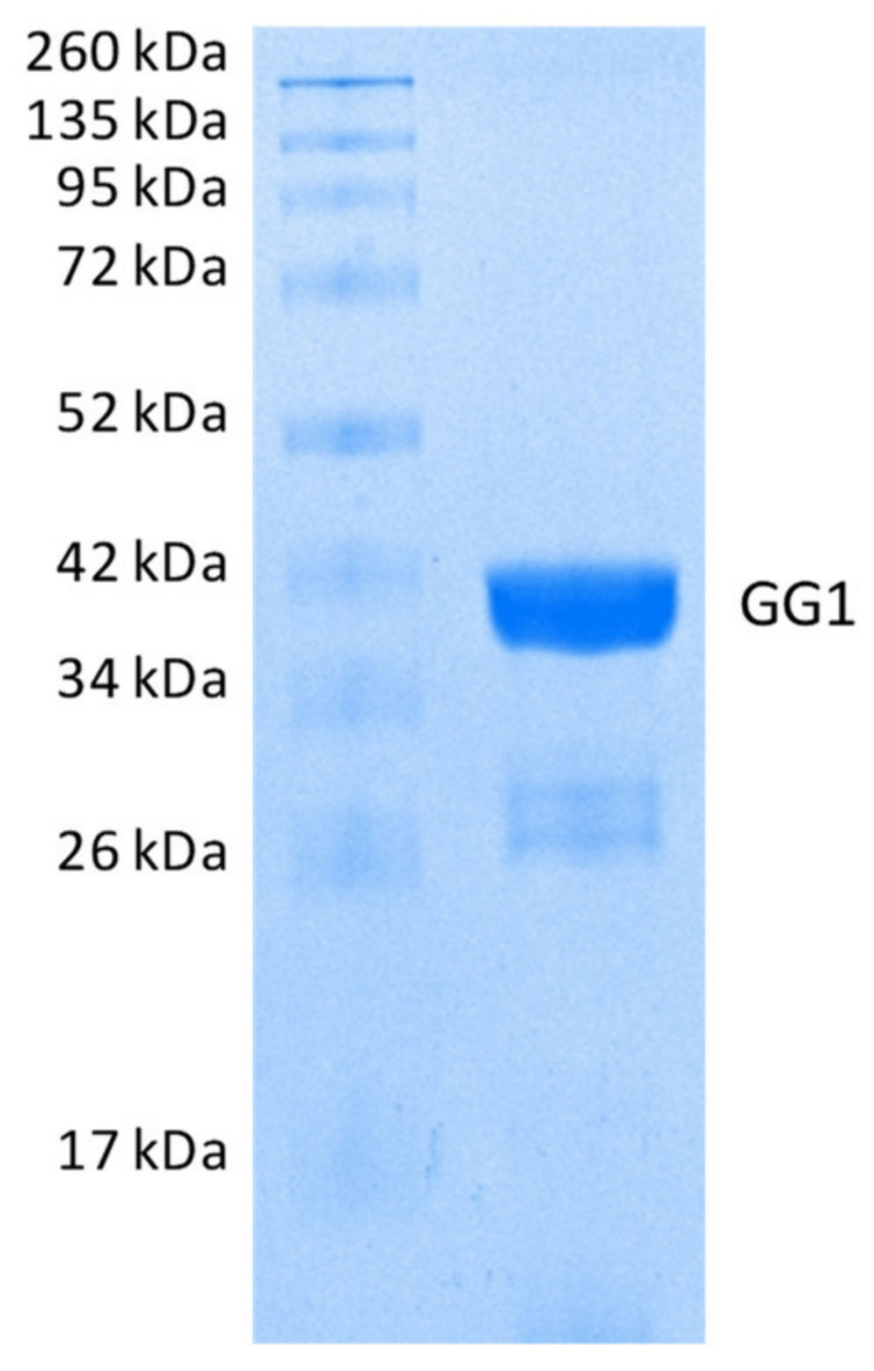
SDS-PAGE of purified GG1 mutant of *Brevundimonas diminuta* PTE. The band of 28 kDa was excised from the gel manually and identified as a degraded form of GG1 by peptide mass fingerprinting using a MALDIT-OF MS Bruker Ultraflex I spectrometer (Bruker Daltonics) after trypsin digestion as previously described [43].

GG1 mutant of *Brevundimonas diminuta* PTE: Multiple mutated PTE (MW = 36,000) was designed to hydrolyze V agents in addition to other OPs. The variant GG1 was previously optimized through ancestral engineering and shown to degrade V-agent surrogates [30]. The gene coding for the variant GG1 from *Brevundimonas diminuta* containing N-terminal Strep-TEV tag was cloned in a pET22b+ vector using NdeI/NotI restriction sites. Heterologous production was performed in *Escherichia coli* BL21 (DE_3_)-pGro7/GroEL (TaKaRa) chaperone expressing strain as previously described. Briefly, production was performed in ZYP medium (10 g/L Tryptone, 5 g/L Yeast Extract, 25 mM (NH_4_)_2_SO_4_, 100 mM KH_2_PO_4_, 50 mM Na_2_HPO_4_, 0.5% (*w/v*) glycerol, 0.05% (*w/v*) glucose 25 g, 0.2% (*w/v*) α-lactose, 100 µg/mL ampicillin and 34 µg/mL chloramphenicol) at 37 °C and 140 rpm shaking. When OD_600nm_ reached 0.8–1, CoCl_2_ (final concentration 0.2 mM) and L-arabinose (final concentration 2 g∙L^−1^) were added, and the temperature was decreased to 16 °C for 16–20 h. Cells were harvested by centrifugation (4400 g, 10 °C, 30 min). Pellets were re-suspended in buffer (50 mM Tris, 300 mM NaCl, pH 8.0). Then, cell lysis was performed by adding DNAseI, lysozyme and PMSF to reach final concentrations of 10 µg∙mL^−1^, 0.25 mg∙mL^−1^ and 0.1 mM, respectively. Subsequently, three steps of 30 s of sonications (Qsonica, Q700; Amplitude 45) were applied in ice. Cell debris were removed by centrifugation (12,000 g, 10 °C, 20 min) and supernatant was filtered (0.8 µM) prior to purification. Purification was performed by affinity chromatography (StrepTrap^TM^ HP GE Healthcare; ÄKTA Avant) and eluted in 50 mM Tris, 300 mM NaCl, 2.5 mM desthiobiotin, pH 8.0. Protein concentration was measured by using a NanoDrop 2000 (Thermo Scientific) spectrophotometer. Then, the protein solution was adjusted to 1 mg/mL with 20 mM Tris buffer, pH 8.0 supplemented with 0.2 mM CoCl_2_. Enzyme purity (85%, corresponding to ([E] = 2.43 × 10^−5^ M) was checked by SDS-PAGE (T = 12.5%) followed by Coomasie Brilliant Blue staining (Figure 5).

### 3.3. Reaction of Probe IV With Thiols and Calibration

“Calbiochem probe IV”, is a coumarinyl derivative that reacts with thiol-chemicals to form a highly fluorescent conjugate. It was found to be the fastest and the most sensitive thiol reagent with a large signal-to-noise ratio [44]. It was, in particular, applied for screening human BChE clones with butyrylthiocholine as a substrate in microfluidic droplets [45]. More recently, Probe IV was used for quantitative measurements of low activity of BChE and AChE and for kinetic analysis of ChE–catalyzed hydrolysis of thioesters [12].

Stock solution of 0.1 M echothiophate was in water and stored at −20 °C. Stock solution of Probe IV was 1mM in DMSO and stored at −20 °C. Because fluorescence of Probe IV-thio-conjugate was found dependent on buffer, 60 μM stock solution of glutathione (GSH), the calibration thiol compound, was in 20 mM Tris/HCl pH 8.0 for calibration of velocity data of GG1 and in 0.1 M sodium phosphate buffer pH 7.0 for calibration of velocity data of G117H mutant. 

Kinetic measurements were performed in a Peltier thermostated spectrofluorimeter F-7100 (Hitachi Ltd., Japan), using standard spectrofluorimetic cuvettes of 1 cm-path length in a total volume of 1.5 mL. The fluorescence emission of Probe IV-thiol conjugate has λ_ex_ = 400 nm and λ_em_ = 465 nm. λ_ex_ slit was 5.0 nm and λ_em_ slit 10.0 nm. Calibration plot was made with glutathione up to 5 μM final in cuvette containing either 0.1 M phosphate buffer pH 7.0 (calibration for G117H activity) or 20 mM Tris/HCl pH 8.0 (calibration for GG1 activity). Fluorescence intensity plot, FI vs [GSH] was built after substraction of the intrinsic fluorescence of Probe IV.

The buffer for the G117H mutant of human ChE was 0.1 M sodium phosphate buffer pH 7.0, and 20 mM Tris/HCl pH 8.0 for GG1. The final concentration of Probe IV in cuvette was 1 μM, and the final DMSO concentration was 1% *v/v*. The final echothiophate concentration in cuvette ranged from 0.05 to 0.8 mM. The final concentration in active sites per assay ranged from 8.51 × 10^−10^ M to 2.85 × 10^−8^ M for G117H mutant and from 1.6 × 10^−8^ to 9.4 × 10^−8^ M for GG1. The temperature for kinetic measurements was set up at 25 °C for the G117H mutant of BChE and at 30 °C for the GG1 mutant of *Brevundimonas diminuta.* PTE.

The rate of GG1-catalyzed hydrolysis (ΔIF/dt) of echothiophate was monitored for 8 min, while for G117H mutant of BChE it was 2 min. The fluorescence background of Probe IV was substracted, as well as the fluorescence background due to spontaneous hydrolysis of echothiophate.

### 3.4. Steady-State Kinetic Study of Enzyme-Catalyzed Hydrolysis of Echothiophate

Steady-state kinetic measurements of initial velocity for enzyme-catalyzed hydrolysis of echothiophate were repeated in triplicate for each enzyme concentration used. Values of ΔF/min were converted in μmol of thiocholine released/min using the GSH calibration plot. 

Previously reported results for G117H showed that at least up to 1 mM echothiophate, the enzyme obeys the Michaelis–Menten kinetic model [10,11]. Also, all reported studies performed on wild type and mutants of *Brevundimonas diminuta* showed that these enzymes obey the Michaelis–Menten model regardless the substrate [39]. Thus, catalytic parameters *V_max_* and *K_m_* were determined by non-linear fitting of initial velocity to the Michaelis–Menten rate equation (Equation (2)), using Origin (OriginLab Co, Northampton, MA, USA).

(2) $$v=\frac{k_{\mathrm{cat}}\left[E\right]\left[S\right]}{K_{m}+\left[S\right]}$$

At very low echothiophate concentration, [OP] << *K_m_,* the bimolecular rate constants *k_cat_/K_m_* were also determined by linear regression of Equation (3).

(3) $$v=\frac{k_{\mathrm{cat}}}{K_{m}}\left[E\right]\left[S\right]$$

### 3.5. Possibility of Unwanted Interactions of DMSO and Probe IV With Enzymes

As previously suggested for wild-type BChE [12], the possibility that 1% DMSO, the solvent of Probe IV, could reversibly inhibit the G117H mutant was checked. The enzyme was assayed by the method of Ellman [9] with BTC (0.1, 0.2, and 0.5 mM) as the substrate and DTNB as the chromogene, in the presence of different concentrations of DMSO (0.5%, 1%, 1.5%, and 2% final). The effect of DMSO on the enzyme was analyzed by Dixon and Cornish–Bowden plots [46].

The possibility that Probe IV could be a reversible inhibitor of G117H mutant was checked. Indeed, Probe IV bears structural components in common with certain fluorescent probes that have been recently recognized has reversible inhibitors of BChE [47].

The possibility that Probe IV could alkylate free thiols was investigated. G117H BChE has no solvent exposed cysteine [42]. On the other hand, GG1 has two naturally occurring cysteines and a residue I106 mutated in cysteine (I106C). This later is located in the active site pocket. For this purpose, 0.047 μM GG1 were pre-incubated from 5 to 20 min in dark with 1 μM in 1% DMSO. After the different pre-incubation times, 0.5 nM echothiophate was added and the enzyme activity measured.

### 3.6. Molecular Modeling

In a previous work, we showed that Probe IV does not inhibit significantly human BChE [12]. Thus, to estimate the possibility of reversible inhibition and/or irreversible inhibition of PTE by Probe IV molecular docking of Probe IV was performed on *Brevundimonas diminuta* PTE. It should be noted that wt PTE possesses 2 free Cysteines in its structure, and the GG1 mutant has an additional Cys in its active center (I106C). A reported result on closely related mutants showed that the two natural Cys do not react with DTNB as the thiol probe, and that C106 is also inaccessible to DTNB [16].

Although the different PTEs has common features in their active site, they have different 3D structures (TIM-barrel fold such as *Brevenundimonas diminuta* PTE, pita bread-fold, 6-propeller-fold: [48], our work was expanded to docking of Probe IV to paraoxonase, a 6-propeller-fold enzyme and *Sulfolobus solfataricus,* another PLL- PTE of TIM-barrel type. Docking into active sites of PTEs is challenging due to the presence of bi-cations and their coordination by active site residues, leading to charge redistribution [49]. To overcome this problem, one solution is to use specially developed charge-independent AutoDock4_Zn_ force field [34] for Zn-containing *B. diminuta* PTE. In case of Co and Fe-containing *S. solfataricus* PTE solution is to use QM-derived partial charges on active site atoms. Due the protein size, structure optimization requires combined QM/MM method [50].

The structure of Probe IV was quantum-mechanically optimized with Gamess-US [51] software (B3LYP/6-31G*). According to previous findings [33], partial atomic were charges assigned according to the Löwdin scheme [52] from quantum mechanics data was used for molecular docking.

X-ray structures of G117H BChE (PDB ID 2XMD [42]); *Brevundimonas diminuta* PTE (PDB ID 1I0D [53] with both Zn^2+^ cations) and GG1 mutant model based on it; PON-1 (PDB ID 1V04 [54]); *Sulfolobus solfataricus* PTE (PDB ID 3UF9 [55]) were used. All water and co-crystallized molecules were removed from all structures; active site bi-cations were kept in PTEs. Hydrogen atoms were added using Reduce software [56].

**Figure 6:**
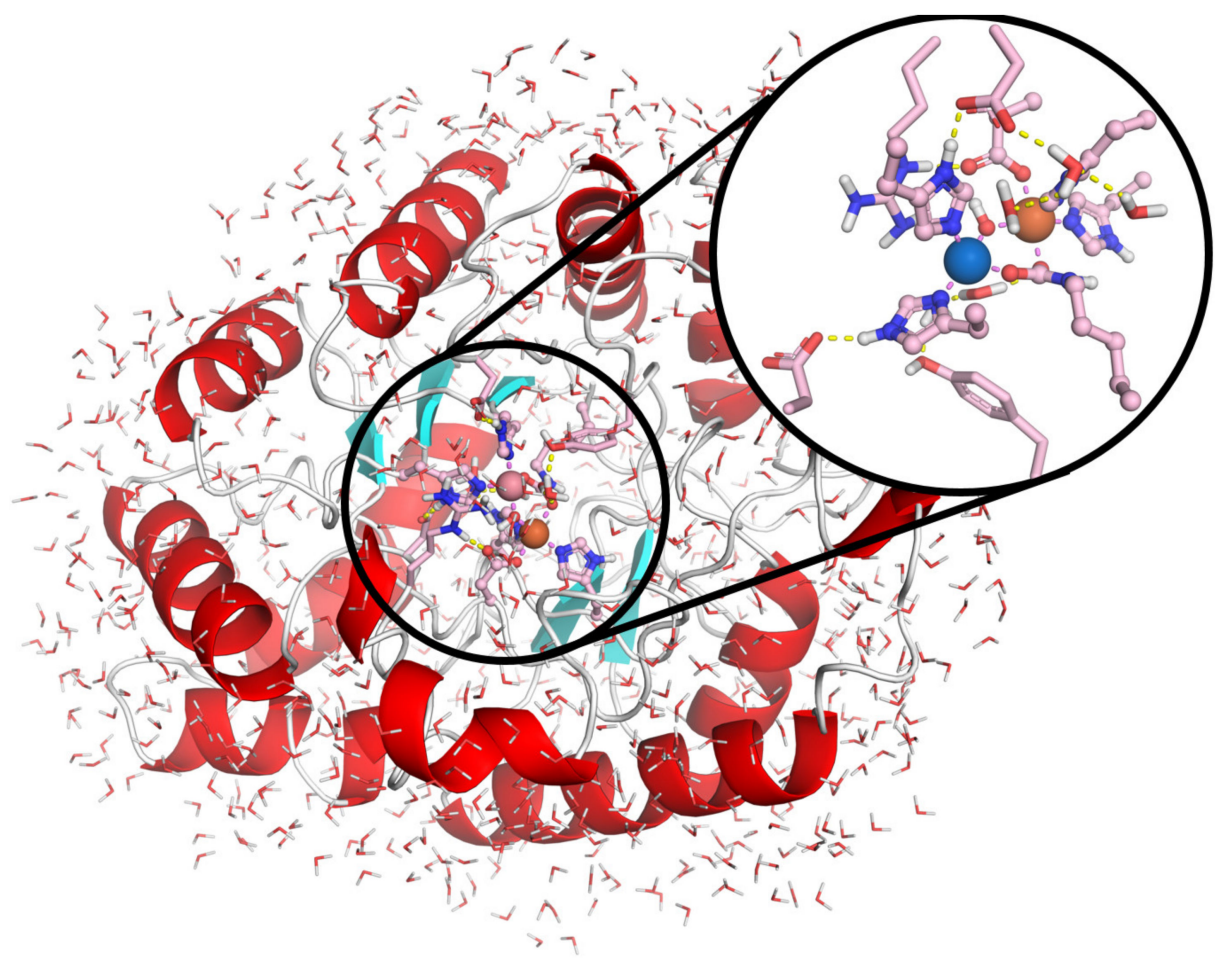
Full *Sulfolobus solfataricus* PTE structure in solvation shell used for QM/MM modeling, QM subsystem with active site is shown in the circle.

For QM/MM optimization of *Sulfolobus solfataricus* PTE, we proceeded as follows: protein was surrounded with 1006 water molecules, forming solvation shell, total size of the system was 8059 atoms (Figure 6). Active site residues and 7 water molecules were included into quantum subsystem 137 atoms total). NwChem 6.5 [57] software was used (PBE0-D3/cc-pvdz/Amber). The effective core potentials (ECPs) with LanL2DZ basis set were used to describe Co^2+^ and Fe^2+^ cations. For optimized active site geometry, partial atomic charges were derived in the same fashion as for the ligand.

For molecular docking AutoDock 4.2.6 software [58] was used with AutoDock4_Zn_ force field for *B. diminuta* PTE [34]. For all proteins grid boxes were 22.5 Å × 22.5 Å × 22.5 Å size (grid spacing 0.375 Å) and embraced active sites of the enzymes. The main Lamarckian Genetic Algorithm (LGA) [59] parameters were set as follows: 256 runs, 25 × 10^6^ evaluations, 27 × 10^4^ generations, and a population size of 3000.

## 4. Conclusions

The simplicity, accuracy, and sensitivity of Probe IV-based spectrofluorimetric assay for investigating steady-state kinetics of enzyme catalyzed hydrolysis of echothiophate as a P–S bonded organophosphorus model make this new analytical method of great interest for research of novel OP hydrolases capable of hydrolyzing V-agents and easy determination of their catalytic properties. In particular, it could advantageously be used for high throughput direct microfluidic screening of recombinant OP hydrolase clones [45]. The high sensitivity of Probe IV is an asset to screen low activities at the beginning of directed evolution cycles. Thus, Probe IV-based assays would be particularly suitable for rapid screening of evolved enzyme libraries, enzyme mining in various biotopes, and for enzyme-based biosensors with improved detection limit of compounds of interest. However, possible covalent binding and non-covalent interactions of Probe IV with enzymes may affect the catalytic parameters of OP hydrolases. Thus, quantitative studies must be supported by thorough molecular modeling and non-spectro(fluorimetric) determinations of binding affinity, e.g., isothermal calorimetry, as well as kinetic study of the reactivity of free cysteines present in enzyme structures. In particular, it should be demonstrated that concentrations of the thiol probe used in activity assays does not affect the measured values of P–S bonded OP hydrolyzing enzyme activity.
