# Correction: Zueva et al. Steady-State Kinetics of Enzyme-Catalyzed Hydrolysis of Echothiophate, a P–S Bonded Organophosphorus as Monitored by Spectrofluorimetry. *Molecules* 2020, *25*, 1371

**DOI:** 10.3390/molecules31122056

**Published:** 2026-06-12

**Authors:** Irina V. Zueva, Sofya V. Lushchekina, David Daudé, Eric Chabrière, Patrick Masson

**Affiliations:** 1 Arbuzov Institute of Organic and Physical Chemistry, Federal Research Center “Kazan Scientific Center of the Russian Academy of Sciences”, Arbuzov str. 8, 420088 Kazan, Russia; zueva.irina.vladimirovna@gmail.com; 2 Emanuel Institute of Biochemical Physics, Russian Academy of Sciences, Kosygin str 4, 119334 Moscow, Russia; sofya.lushchekina@gmail.com; 3 Gene&GreenTK, HU Méditerranée Infection, Jean Moulin Blvd 19–21, 13005 Marseille, France; david.daude@gene-greentk.com; 4 Aix-Marseille University, IRD, APHM, MEPHI, IHU-Méditerranée Infection, 15005 Marseille, France; eric.chabriere@univ-amu.fr; 5 Kazan Federal University, Neuropharmacology Laboratory, Kremlevskaya str 18, 480002 Kazan, Russia

In the original publication [1], the Conflicts of Interest statement of David Daudé and Eric Chabrière were not included. The updated Conflicts of Interest should be as follows:

David Daudé and Eric Chabrière have filed the patent WO2019016468. David Daudé and Eric Chabrière report receiving personal fees from Gene&GreenTK during the study. David Daudé and Eric Chabrière are shareholders in Gene&GreenTK. David Daudé is CEO of Gene&GreenTK. The company, Gene&GreenTK, had no role in the study design, collection, analysis, interpretation of data, the writing of this article or the decision to publish the results. All other authors declare no competing interests.

The authors state that the scientific conclusions are unaffected. This correction was approved by the Academic Editor. The original publication has also been updated.
